# Android Fat Depot Is More Closely Associated with Metabolic Syndrome than Abdominal Visceral Fat in Elderly People

**DOI:** 10.1371/journal.pone.0027694

**Published:** 2011-11-11

**Authors:** Seon Mee Kang, Ji Won Yoon, Hwa Young Ahn, So Yeon Kim, Kyoung Ho Lee, Hayley Shin, Sung Hee Choi, Kyong Soo Park, Hak Chul Jang, Soo Lim

**Affiliations:** 1 Department of Internal Medicine, Seoul National University College of Medicine and Seoul National University Bundang Hospital, Seongnam, Korea; 2 Department of Radiology, Seoul National University College of Medicine and Seoul National University Bundang Hospital, Seongnam, Korea; 3 Johns Hopkins Bloomberg School of Public Health, Baltimore, Maryland, United States of America; 4 Department of Internal Medicine, Seoul National University College of Medicine, Seoul, Korea; University of Padova, Italy

## Abstract

**Background:**

Fat accumulation in android compartments may confer increased metabolic risk. The incremental utility of measuring regional fat deposition in association with metabolic syndrome (MS) has not been well described particularly in an elderly population.

**Methods and Findings:**

As part of the Korean Longitudinal Study on Health and Aging, which is a community-based cohort study of people aged more than 65 years, subjects (287 male, 75.9±8.6 years and 278 female, 76.0±8.8 years) with regional body composition data using Dual energy X-ray absorptiometry for android/gynoid area, computed tomography for visceral/subcutaneous adipose tissue (VAT/SAT), and cardiometabolic markers including adiponectin and high-sensitivity CRP were enrolled. We investigated the relationship between regional body composition and MS in multivariate regression models. Mean VAT and SAT area was 131.4±65.5 cm^2^ and 126.9±55.2 cm^2^ in men (P = 0.045) and 120.0±46.7 cm^2^ and 211.8±65.9 cm^2^ in women (P<0.01). Mean android and gynoid fat amount was 1.8±0.8 kg and 2.5±0.8 kg in men and 2.0±0.6 kg and 3.3±0.8 kg in women, respectively (both P<0.01). VAT area and android fat amount was strongly correlated with most metabolic risk factors compared to SAT or gynoid fat. Furthermore, android fat amount was significantly associated with clustering of MS components after adjustment for multiple parameters including age, gender, adiponectin, hsCRP, a surrogate marker of insulin resistance, whole body fat mass and VAT area.

**Conclusions:**

Our findings are consistent with the hypothesized role of android fat as a pathogenic fat depot in the MS. Measurement of android fat may provide a more complete understanding of metabolic risk associated with variations in fat distribution.

## Introduction

Obesity is a heterogeneous disorder characterized by multi-factorial etiology. Obese individuals vary in their body fat distribution, their metabolic profile and the degree of associated cardiovascular and metabolic risks. There is substantial evidence providing that fat distribution is a better predictor of cardiovascular disease than the degree of obesity [1]–[5]. An excess of abdominally located fat, even without manifestations of obesity, is associated with metabolic disturbances that indicate an increased risk of atherogenesis and of higher morbidity and mortality, possible due to inherent characteristics of abdominal adipocytes [3], [4], [6], [7]. Thus, regional fat distribution rather than overall fat volume has been considered to be more important in understanding the link between obesity and metabolic disorders. Among fat depots, fat accumulation in the abdominal area has a greater risk of developing diabetes and future cardiovascular events than the peripheral area [8]. There are differences between adipose tissue present in subcutaneous areas and in the abdominal cavity. These include anatomical, cellular, molecular, physiological, clinical and prognostic differences [2], [7], [9]. Many studies have suggested that visceral adipose tissue (VAT) compared with subcutaneous adipose tissue (SAT) is more cellular, vascular and innervated with a larger number of inflammatory and immune cells, lesser preadipocyte differentiating capacity, and a greater percentage of large adipocytes [9]. Similar findings were also observed across different races/ethnicities including Japanese where an independent association with VAT was found even after accounting for multiple risk factors [7]. Therefore, fat distribution rather than its magnitude may be more significant in understanding metabolic risk, particularly the varying impacts of VAT and SAT.

In a different context, truncal fat depot can be partitioned into upper body (android or central) and lower body (gynoid or peripheral) area. Empirically, android or central fat deposition is known to be more associated with cardiometabolic risk than gynoid or peripheral fat deposition. Many studies with simple anthropometric measurements such as waist circumference or waist-to-hip ratio have given more weight to the central adiposity [6], [10]–[12]. More advanced technology with computed tomography (CT) or dual energy X-ray absorptiometry (DXA) has been used to measure the regional fat mass. CT has an advantage in distinguishing between VAT and SAT while DXA can measure compartment body compositions such as android and gynoid area. However, there are limited studies investigating the implication of android/gynoid fat deposition assessed by advanced technology in determining cardiometabolic risks.

Metabolic syndrome (MS) increases cardiovascular morbidity and mortality, and all cause of mortality [13]. MS also increases the risk of developing diabetes mellitus with its components representing major risk factors for impaired glucose metabolism [14]. Obesity, particularly abdominal obesity, is a key feature of a cluster of atherothrombotic and inflammatory abnormalities associated with MS [15]. There is substantial evidence linking central obesity with cardiovascular disease and the other MS components as well as its critical role in the etiological cascade leading to full-blown manifestations of MS.

Thus, assessment of fat distribution may be important in the clinical evaluation of cardiometabolic risks. However, there has been no comprehensive study on fat distribution related risks particularly in elderly Asian populations whose physical and metabolic characteristics differ from those of Caucasians. We evaluated the association between clustering of components constituting MS and the whole and regional body composition measured by comprehensive methods including DXA and CT in a community-based cohort study of elderly men and women. The effects of metabolic or inflammatory markers were also evaluated.

## Methods

### Subjects, anthropometric and biochemical parameters

This study was part of the Korean Longitudinal Study on Health and Aging (KLoSHA), which is a cohort that began in 2005 and consisted of 1000 Korean subjects aged over 65 years (439 men and 561 women) recruited from Seongnam city, one of the satellites of Seoul Metropolitan district. The study population and part of the method of measurements for the cohort have been published previously [16].

The current study subjects were from the KLoSHA. Of the original 1000 KLoSHA subjects, we randomly selected 600 participants (60% of the KLoSHA subjects) for assessment of body composition. Of these 600 subjects, 21 declined the DXA or CT scans and 14 were unable to undergo the examination due to their poor physical condition. In total, 565 participants (94.2% of 600 selected subjects) who underwent DXA/CT scans for body composition evaluation were enrolled in the current analysis. Pertinent demographic and other characteristics of the selected subjects were similar to the cohort population. Among study participants, 39.1% (n = 221) were found to have diabetes: 17.5% (n = 99) were previously on antidiabetic medication and 21.6% (n = 122) were diagnosed with diabetes by 75 g standard OGTT which was performed as s study screening procedure. Smoking and alcohol status was divided into three categories; current smoker, ex-smoker, or never smoker, and current drinker, ex-drinker, or never drinker, respectively. Current drinker was defined as a person consuming more than 4 drinks/week (50 g/day of ethanol). Physical activity was divided into two categories; none or regular exercise. Regular exercise was defined as exercising more than three times a week (each session should be at least 30 min long). The homeostasis model assessment of the insulin resistance (HOMA-IR) was calculated as reported previously [17]. Several metabolic markers including adiponectin and high-sensitivity CRP (hsCRP) which are known to be associated with MS were measured. Detailed information about measurement method was published previously [16]. MS was defined according to the NCEP guideline as the presence of at least three of the following components: abdominal obesity (waist circumference ≥90 cm for men and ≥80 cm for women), triglyceride level ≥150 mg/dL, high-density lipoprotein cholesterol level <40 mg/dL for men and <50 mg/dL for women, blood pressure ≥135/85 mmHg, and/or fasting blood glucose level ≥110 mg/dL [18]. All the assessments were performed at Seoul National University Bundang Hospital (SNUBH). This was approved by the Institutional Review Board of SNUBH. The written, informed consent for subjects undergoing CT procedure to inform them of radiation hazard and possible contrast toxicity was obtained from each individual as a routine procedure.

### Regional body composition by DXA

DXA measures were recorded using a bone densitometer (Lunar, GE Medical systems, Madison, WI). DXA is quantified by body tissue absorption of photons that are emitted at two energy levels to resolve body weight into bone mineral, lean and fat soft tissue masses. In vivo precision for body composition measurements using DXA was proven previously [19]. In this study, precision was excellent for lean tissue mass (root mean square of 0.21 kg; coefficient of variation (CV) of 0.4%) and precision error for the total fat mass had a CV 0.86% and 0.88% for the percentage fat. A high correlation between consecutive measurements was observed for all three compartment body compositions including total body bone mineral content, lean mass, and fat mass (standard error of = 0.993−1.002; all r^2^ = 0.99).

**Figure 1:**
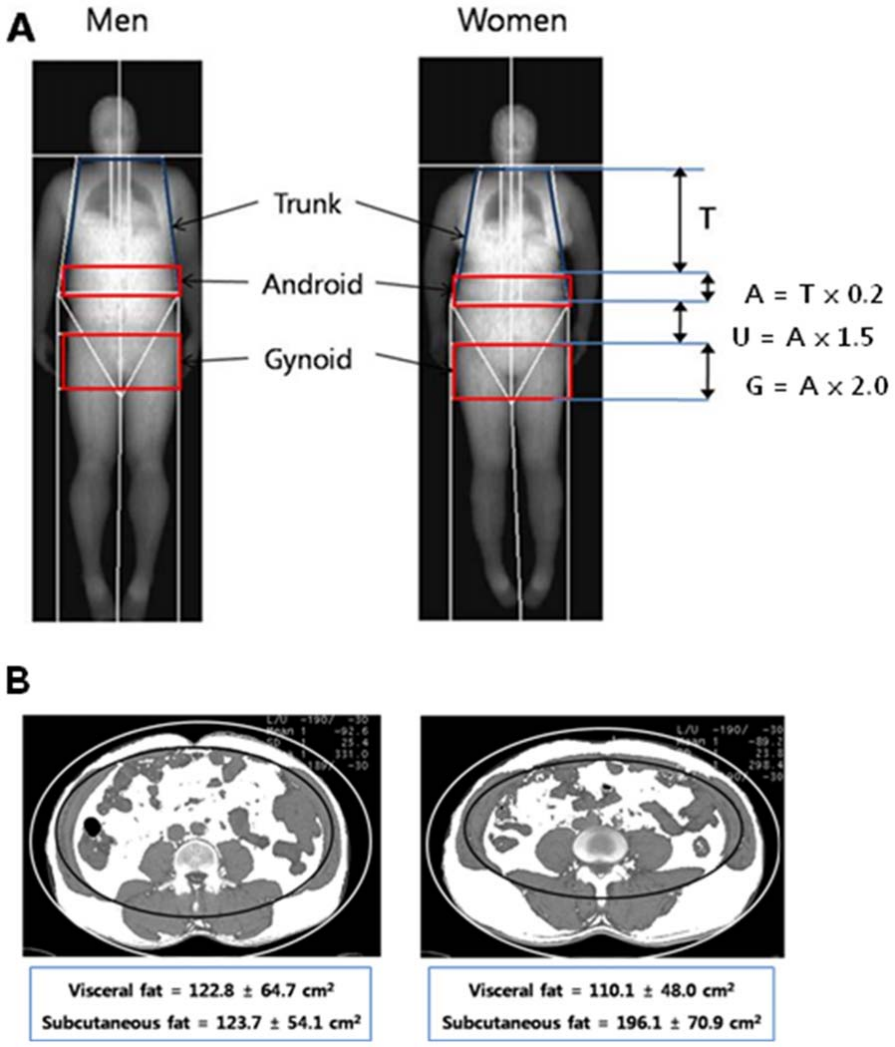
Regional body composition measurement by DXA (A) and CT (B).

The regions of interest (ROI) for regional body composition were defined using the software provided by the manufacturer (**Figure 1A**):

- Trunk ROI (T): from the pelvis cut (lower boundary) to the neck cut (upper boundary).
- Android fat distribution ROI (A): from the pelvis cut (lower boundary) to above the pelvis cut by 20% of the distance between the pelvis and neck cuts (upper boundary).
- Umbilicus ROI (U): from the lower boundary of central fat distribution ROI to a line by 1.5 times the height of the android fat distribution ROI (lower boundary).
- Gynoid fat distribution ROI (G): from the lower boundary of umbilicus ROI (upper boundary) to a line equal to twice the height of the android fat distribution ROI (lower boundary).

### Abdominal (visceral and subcutaneous) fat areas by CT

CT scans were obtained using a 64–detector (Brilliance; Philips Medical Systems, Cleveland, Ohio). All patients were placed in the supine position and were scanned from L3-4 to L5-S1 intervetebral disc level. The tube voltage was 120 kVp for 64 detector row scanner. Effective tube current-time product generally ranged between 20–50 mAs. The images were reconstructed with 5 mm thickness with 5 mm-intervals. One slice obtained at the level of umbilicus were selected and the amount of the total abdominal fat were calculated by measuring the area of the pixels whose attenuation values ranged from −190 to −30 Hounsfield unit (HU) using a commercially available software (Rapidia, version 2.8, Infinitt Co., Seoul) [20]. VAT was defined as fat area confined to the abdominal wall musculature. After subtracting VAT from total fat area, the remainder was defined as SAT (**Figure 1B**).

### Cardiac CT angiography to assess coronary artery stenosis

Detailed information about the cardiac CT angiography protocol was described previously [21]. Briefly, CT angiography was performed with a 64-slice multidetector-row cardiac CT scanner (Brilliance 64; Philips Medical Systems, Best, The Netherlands), and a standard scanning protocol was used [21]. All scans were analyzed independently in a blind fashion using a three-dimensional workstation (Brilliance; Philips Medical Systems). Each lesion was identified using a multiplanar reconstruction technique and maximum intensity projection of the short axis, in two-chamber and four-chamber views. Coronary artery lesions were analyzed according to the modified American Heart Association classification [22].

### Statistical analyses

All data are presented as the mean and SD or n and %, and were analyzed using SPSS Windows version 11.0 (SPSS Inc., Chicago, IL). The demographic and laboratory characteristics of subjects were compared using Student's t test or a Chi-square test according to the presence of MS. Correlations between variables were analyzed using Pearson's correlation. Multiple regression analysis was used to determine the independent effect of body composition parameters on clustering of five components of MS. P<0.05 was considered significant.

## Results

Anthropometric, body composition, and metabolic characteristics of the study population stratified by sex are provided in **Table S1**. Men (n = 287) and women (n = 278) in our study were of similar age. Mean age (± SD) of study subjects was 73.6±7.6 years for men and 72.5±6.7 years for women. BMI (± SD) was 24.1±3.2 kg/m^2^ for men and 24.6±3.1 kg/m^2^ for women. Men were more likely to have unfavorable lifestyle habits including smoking and alcohol consumption, nevertheless the proportion of participants who engaged in regular exercise was significantly higher in men than in women. The concentrations of HDL- and LDL-cholesterol, and adiponectin were significantly greater in women whereas fasting plasma glucose concentration were higher in men. There was no significant difference in the concentration of triglycerides, fasting insulin, A1C, and hsCRP levels between men and women. Whole body muscle mass measured by DXA was significantly greater in men. Whole body fat mass, android and gynoid fat amount measured by DXA, and SAT quantified by CT were significantly higher in women than men. In contrast, VAT quantified by CT was greater in men than in women (P<0.05).

### Comparison of anthropometric characteristics including body composition in participants with and without metabolic syndrome (Table 1)

**Table 1:** Participants characteristics including body composition measured by dual energy x-ray absorptiometry (DXA) and computed tomography (CT).

	No MS (n = 268)		MS (n = 297)		P-value
	Mean	SD	Mean	SD	
Age (years)	72.5	6.9	73.6	7.4	0.067
Male (n, %)	159	59.3%	128	43.1%	<0.001
SBP (mmHg)	128.9	17.7	136.6	16.6	<0.001
DBP (mmHg)	81.6	11.0	85.0	10.4	<0.001
BMI (kg/m^2^)	23.1	3.0	25.5	2.9	<0.001
Waist circumference (cm)	82.8	9.0	90.3	7.9	<0.001
Smoking					0.037
Current smoker (n, %)	37	13.8%	32	10.8%	
Ex-smoker (n, %)	89	33.2%	76	25.6%	
Never smoker (n, %)	142	53.0%	189	63.6%	
Alcohol					0.008
Current drinker (n, %)	89	33.5%	66	22.3%	
Ex-drinker (n, %)	41	15.4%	44	14.9%	
Never drinker (n, %)	136	51.1%	186	62.8%	
Regular exercise (n, %)	166	62.6%	160	54.4%	0.049
Medication					
Antihypertensive medication	89	33.2%	160	53.9%	<0.001
Antidiabetic medication	26	9.7%	73	23.6%	<0.001
Lipid lowering medication	30	11.2%	40	13.5%	0.445
**By DXA**					
Whole body muscle mass (kg)	37.1	7.2	36.9	37.1	0.801
Whole body fat mass (kg)	18.5	7.4	24.3	18.5	<0.001
Android fat mass (kg)	1.6	0.7	2.1	1.6	<0.001
Gynoid fat mass (kg)	2.6	0.9	3.2	2.6	<0.001
**By CT**					
Visceral adipose tissue (cm^2^)	103.9	52.9	149.8	55.4	<0.001
Subcutaneous adipose tissue (cm^2^)	134.2	68.4	189.4	66.8	<0.001
**By cardiac CT angiography**					
Coronary artery stenosis (%)	20.1	21.6	25.6	25.2	<0.001
**Biochemical data**					
Triglycerides (mg/dL)	104.2	41.4	171.6	107.8	<0.001
HDL-cholesterol (mg/dL)	51.1	12.0	40.5	10.4	<0.001
LDL-cholesterol (mg/dL)	129.2	33.9	127.4	35.5	0.532
Fasting glucose (mg/dL)	104.9	20.0	117.5	29.0	<0.001
Fasting insulin (µIU/mL)	4.3	2.1	6.0	3.8	<0.001
HOMA-IR*	1.1	0.6	1.8	1.2	<0.001
A1C (%)	5.9	0.7	6.3	1.0	<0.001
Adiponectin (µg/mL)	10.1	6.1	7.4	5.1	<0.001
hsCRP (mg/dL)	0.3	0.8	0.2	0.5	0.413

*HOMA-IR; homeostasis model assessment for insulin resistance.

Of the study population of 565 elderly people (73.0±7.2 years of age), 47.4% (n = 268) fulfilled the criteria of MS. Participants with or without MS were similar in age, but more women had MS than men. Systolic and diastolic blood pressure, BMI, and waist circumference were significantly higher in participants with MS compared to without MS. In terms of specific adiposity measurements, whole body fat mass, total android and gynoid tissue, android and gynoid fat amount measured by DXA, and VAT and SAT quantified by CT scan were all greater in participants with MS compared to without MS. The concentrations of triglycerides, and HDL-cholesterol, fasting glucose and insulin, and A1C levels, and HOMA-IR were significantly higher in participants with MS than without MS. Circulating adiponectin levels were significantly lower in participants with MS, whereas hsCRP level was not significantly different between two groups. In terms of lifestyle habits, the proportion of subjects with cigarette smoking and alcohol consumption were significantly higher in MS. However participants with MS were more likely to engage in regular exercise. Past medical history of coronary heart disease (i.e. angina, myocardial infarction, percutaneous coronary intervention, and coronary artery bypass surgery) or strokes were not different.

### Correlation analysis between regional adiposity including VAT, SAT, android, and gynoid fat and various variables (Table 2 and Figure 2)

**Figure 2:**
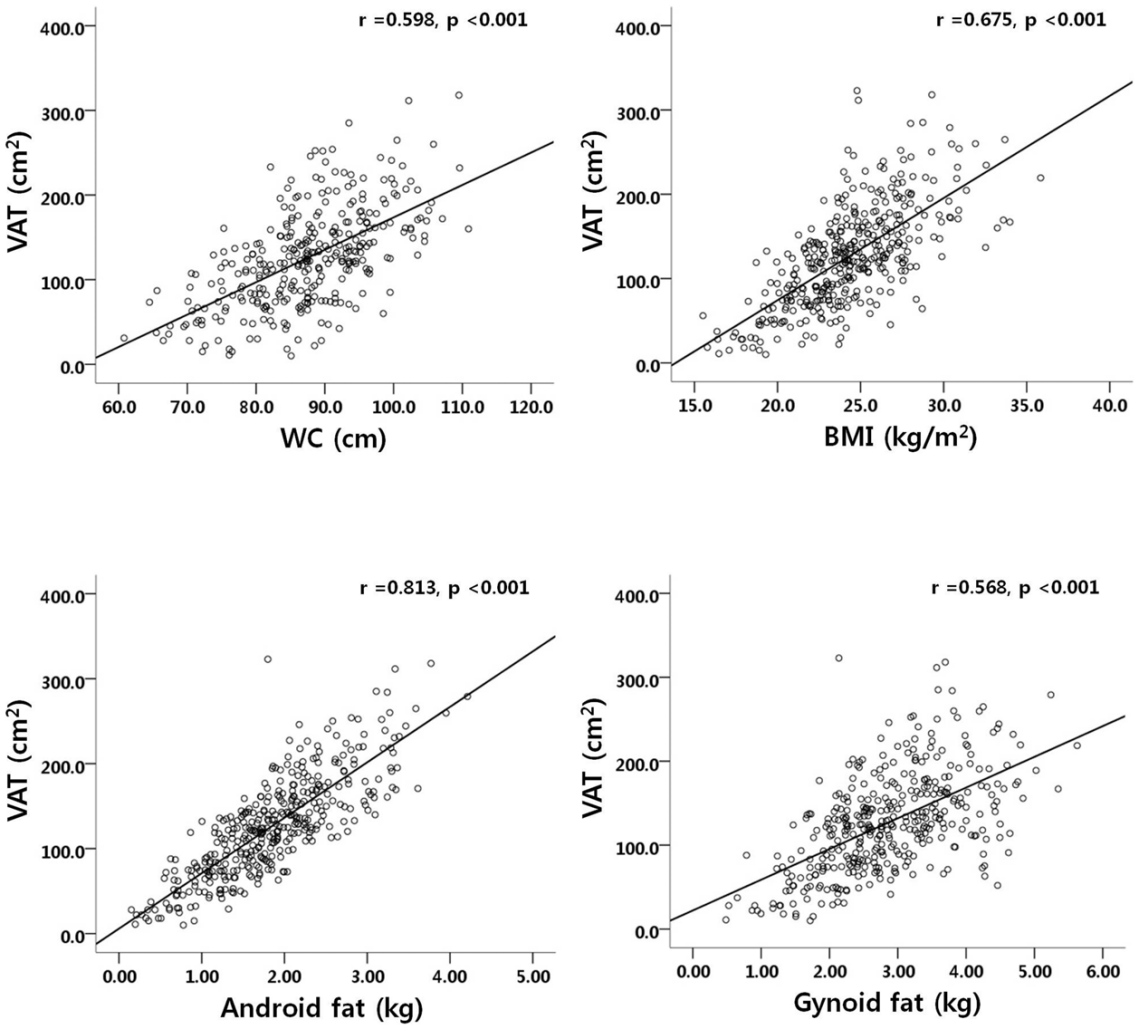
Association between waist circumference (WC), body mass index (BMI), android and gynoid fat measured by DXA, and visceral adipose tissue (VAT) measured by CT.

**Table 2:** Correlation analysis between adiposity indices including visceral and subcutaneous adipose tissue (VAT and SAT) measured by CT and android and gynoid fat measured by DXA with various variables.

	VAT	SAT	Android fat	Gynoid fat
Age (years)	−0.078	−0.111	−0.128*	−0.167*
BMI (kg/m^2^)	0.675**	0.649**	0.773**	0.697**
Waist circumference (cm)	0.598**	0.438**	0.661**	0.450**
**By DXA**				
Whole body muscle mass (kg)	0.314**	−0.288**	0.169*	−0.111*
Whole body fat mass (kg)	0.696**	0.809**	0.927**	0.945**
Android fat mass (kg)	0.813**	0.684**	1	0.797**
Gynoid fat mass (kg)	0.568**	0.794**	0.797**	1
Android/gynoid fat ratio	0.624**	0.163**	0.594**	0.032
**By CT**				
VAT (cm^2^)	1	0.442**	0.813**	0.568**
SAT (cm^2^)	0.442**	1	0.684**	0.794**
VAT/SAT	0.544**	−0.413**	0.159**	−0.137*
**By cardiac CT angiography**				
Coronary artery stenosis	0.225**	−0.098	0.201**	0.033
**Biochemical data**				
Triglycerides (mg/dL)	0.211**	0.169*	0.238**	0.147*
HDL-cholesterol (mg/dL)	−0.284**	−0.049	−0.224**	−0.079
LDL-cholesterol (mg/dL)	0.036	0.108	0.063	0.112**
Fasting glucose (mg/dL)	0.207**	0.074	0.205**	0.035
Fasting insulin (µIU/mL)	0.488**	0.414**	0.478**	0.391**
HOMA-IR	0.514**	0.400**	0.509**	0.362**
A1C (%)	0.205**	0.120	0.244**	0.067
Adiponectin (µg/mL)	−0.346**	−0.110	−0.276**	−0.092
hsCRP (mg/dL)	0.075	−0.057	0.023	0.006

**Correlation is significant at the 0.01 level (2-tailed).

There was a negative correlation between age and android and gynoid fat amount (both P<0.01). BMI and waist circumference were highly correlated with VAT and SAT, and android and gynoid fat amount (all P<0.01). VAT at the level of umbilicus was significantly correlated with adiposity measurements by DXA including whole body fat mass, android and gynoid fat amount. However, the correlation coefficient was significantly greater between VAT and android fat than between VAT and gynoid fat (P<0.05). The concentration of triglycerides was associated with all of the four adiposity indices including VAT and SAT, and android and gynoid fat amount whereas HDL-cholesterol showed negative association with adiposity indices. Android fat amount was associated with fasting glucose and insulin levels, HOMA-IR, and A1C, whereas gynoid fat was not associated with fasting glucose and A1C levels. Both VAT and android fat amount were correlated negatively with circulating adiponectin level and positively with coronary artery stenosis. Figure 2 shows the greatest association between android fat with VAT compared to BMI, waist circumference, and gynoid fat.

### Correlation between various parameters including body composition and summation of components of MS

Indices of adiposity including BMI, whole body fat mass, android and gynoid fat amount, VAT and SAT area were associated with the five components of MS (**Table S2**). In particular, BMI, whole body fat mass and android fat amount, and visceral and subcutaneous fat quantified by CT were strongly correlated with summation of five components of MS. Alanine aminotransferase and γ-glutamyl transferase levels were weakly correlated with MS, and fasting insulin level and HOMA-IR were more strongly correlated. Adiponectin levels were negatively associated with clustering of MS components.

### Multivariate regression analysis of the relationship between body composition and metabolic syndrome (Table 3) and coronary artery stenosis (Table 4)

**Table 3:** Multivariate linear regression analysis of associations of multiple parameters including body composition with summation of five individual components of metabolic syndrome.

	β coefficient	t	P-value
Model 1: Age, gender, smoking, exercise, BMI, hsCRP, LDL-cholesterol, adiponectin, HOMA-IR, and whole body fat mass adjusted			
Age (years)	0.150	4.136	<0.001
Gender (male vs. female)	0.210	4.229	<0.001
BMI (kg/m^2^)	0.211	3.429	0.001
hsCRP (≥ 2.5 mg/l vs. <2.5 mg/l)	0.187	3.012	0.034
Adiponectin (µg/mL)	−0.225	−6.002	<0.001
HOMA-IR	0.200	4.850	<0.001
Whole body fat mass (kg)	0.114	1809	0.071
Model 2: Model 1+VAT			
Age (years)	0.121	2.743	0.006
Gender (male vs. female)	0.276	4.527	<0.001
BMI (kg/m^2^)	0.143	1.869	0.062
hsCRP (≥ 2.5 mg/l vs. <2.5 mg/l)	0.156	2.891	0.041
Adiponectin (µg/mL)	−0.226	−4.852	<0.001
HOMA-IR	0.178	3.412	0.001
VAT (cm^2^)	0.172	2.493	0.013
Model 3: Model 1+android fat			
Age (years)	0.143	3.965	<0.001
Gender (male vs. female)	0.275	5.204	<0.001
BMI (kg/m^2^)	0.207	3.399	0.001
hsCRP (≥ 2.5 mg/l vs. <2.5 mg/l)	0.142	2.528	0.063
Adiponectin (µg/mL)	−0.194	−5.094	<0.001
HOMA-IR	0.173	4.153	<0.001
Whole body fat mass (kg)	−0.243	−1.976	0.049
Android fat (kg)	0.384	3.381	0.001
Model 4: Model 1+VAT+android fat			
Age (years)	0.119	2.712	0.007
Gender (male vs. female)	0.317	5.032	<0.001
BMI (kg/m^2^)	0.151	1.976	0.049
Adiponectin (µg/mL)	−0.203	−4.298	<0.001
HOMA-IR	0.159	3.043	0.003
Android fat (kg)	0.378	2.404	0.017

HOMA-IR: homeostasis model assessment for insulin resistance.

**Table 4:** Multivariate linear regression analysis of associations of multiple parameters including body composition with coronary artery stenosis.

	β coefficient	t	P-value
Model 1: Age, gender, smoking, exercise, BMI, hsCRP, LDL-cholesterol, adiponectin, HOMA-IR, and whole body fat mass adjusted			
Age (years)	0.237	4.308	<0.001
Gender (male vs. female)	0.106	1.929	0.055
hsCRP (≥ 2.5 mg/l vs. <2.5 mg/l)	0.187	3.012	0.034
Adiponectin (µg/mL)	−0.152	−2.009	0.046
Whole body fat mass (kg)	0.114	1.809	0.071
Model 2: Model 1+VAT			
Age (years)	0.262	3.726	<0.001
Gender (male vs. female)	0.109	1.927	0.089
hsCRP (≥ 2.5 mg/l vs. <2.5 mg/l)	0.156	2.891	0.041
Adiponectin (µg/mL)	−0.226	−4.852	<0.001
VAT (cm^2^)	0.162	2.321	0.018
Model 3: Model 1+android fat			
Age (years)	0.237	4.288	<0.001
Gender (male vs. female)	0.105	1.884	0.060
hsCRP (≥ 2.5 mg/l vs. <2.5 mg/l)	0.142	2.528	0.056
Adiponectin (µg/mL)	−0.294	−5.094	<0.001
Android fat (kg)	0.159	2.312	0.026
Model 4: Model 1+VAT+android fat			
Age (years)	0.247	3.472	0.001
Gender (male vs. female)	0.123	1.993	0.042
hsCRP (≥ 2.5 mg/l vs. <2.5 mg/l)	0.102	1.528	0.063
Adiponectin (µg/mL)	−0.158	−2.087	0.038

HOMA-IR: homeostasis model assessment for insulin resistance.

Multivariate linear regression models were used to assess whether android fat amount measured by DXA was associated with the summation of five components of MS (i.e. central obesity, hypertension, high triglyceride and low HDL-cholesterol, dysglycemia) controlling for VAT quantified by CT. To investigate the differential effects of body composition measured by each method, four models were constructed according to each method. In Model 1, age, gender, smoking status, exercise habit, BMI, hsCRP (≥ 2.5 mg/l vs. <2.5 mg/l), LDL-cholesterol, adiponectin, HOMA-IR, and whole body fat mass were selected as independent variables. In Model 2, VAT area was added as an independent variable. In Model 3, android fat was further added to Model 1 as an independent variable. Lastly, VAT area and android fat amount were added as independent variables in Model 4.

In model 1, age, female gender, BMI, hsCRP and HOMA-IR were positively associated with clustering of MS components, whereas adiponectin was negatively associated. Adjusting for VAT resulted in a positive association of MS with age, female gender, hsCRP, HOMA-IR, and VAT, and a negative association with adiponectin (Model 2). Association with BMI was attenuated after including VAT in the model. Adjusting for android fat with MS, age, gender, BMI, HOMA-IR, and android fat were positively associated with MS, and negatively associated with adiponectin (Model 3). Finally, adjusting for both VAT and android fat in Model 4 yielded a consistent and unchanged positive association of android fat with MS, whereas an association with VAT was attenuated. When the combined VAT area between L3-4 and L5-S1 was used instead of a single level of VAT (992.3±48.7 cm^2^ in men and 1469.4±53.7 cm^2^ in women, P<0.001), this merged VAT area was associated with MS with a borderline significance (**Table S3**). Including medication history in the regression analysis did not affect the significant association between android fat/visceral fat and MS.

We further investigated the association between android fat/VAT and coronary artery stenosis. In univariate analysis, android fat and VAT were significantly associated with the degree of coronary artery stenosis. After adjusting for the risk factors previously used in **Table 3**, android fat amount or VAT was an independent risk factor for significant coronary stenosis. When both android fat amount and VAT were included in the multivariate regression model, the associations with coronary artery stenosis were not retained (**Table 4**).

## Discussion

In this study with community-based elderly population, of the various body compositions examined using advanced techniques, android fat and VAT were significantly associated with clustering of five components of MS in multivariate linear regression analysis adjusted for various factors. When android fat and VAT were both included in the regression model, only android fat remained to be associated with clustering of MS components. The results suggest that android fat is strongly associated with MS in the elderly population even after adjusting for VAT.

Abdominal obesity is well recognized as a major risk factor of cardiovascular disease and type 2 diabetes [11]. Although anthropometric measurements such as BMI and waist circumference are widely used to estimate abdominal obesity, distinguishing between visceral and subcutaneous fat or between fat and lean mass cannot be ascertained. Moreover, anthropometric measurements are subject to intra- and inter-examiner variations.

Alternatively, more accurate methods used to measure regional fat depot are DXA and CT. DXA and CT provide a comprehensive assessment of the component of body composition with each contributing its unique advantages. CT can distinguish between visceral and subcutaneous fat, and has been useful in measuring fat or muscle distribution at specific regions [23], [24]. However, there are several limitations in the VAT quantification using CT scan. Even though VAT from a single scan obtained at the level of umbilicus was well correlated with the total visceral volume [25], there could be a potential concern for over- or underestimation if we measure fat area at one selected level instead of measuring total fat volume. In addition, CT scan has a greater risk of radiation hazards than DXA and is not appropriate for repetitive measurements [20], [26].

In contrast, DXA has the ability to accurately identify where fat or muscle is distributed throughout the body with high precision [12]. The measurement of body composition is an area, which has attracted great interest because of the relationships between fat and lean tissue mass with health and disease. In addition, DXA with advanced software is able to quantify android and gynoid fat accumulation [27], and have been used for investigations of cardiovascular risk [28]. Adipose tissue in the android region quantified by DXA has been found to have effects on plasma lipid and lipoprotein concentrations[29] and correlate strongly with abdominal visceral fat [30], [31]. Thus, DXA is emerging as a new standard for body composition assessment due to its high precision, reliability and repeatability [32], [33].

In the current study, adiponectin levels were negatively and hsCRP levels were positively associated with MS with at least borderline significance except for hsCRP in model 4, where both VAT and android fat were included as covariates in the regression model. The close relationship between hsCRP and VAT/android fat may have attenuated the association between hsCRP and MS.

Mechanistically and theoretically, fat deposition in android area is suggested to have deleterious effects on the heart function, energy metabolism and development of atherosclerosis. However, studies on android fat depot are limited [23]. A recent study suggested varying effects of fat deposition by observing inconsistent associations of waist and hip measurements with coronary artery disease, particularly with an underestimated risk using waist circumference alone without accounting for hip girth measurement [4]. A more recent study demonstrated that central fat based on simple anthropometry was associated with an increased risk of acute myocardial infarction in women and men while peripheral subcutaneous fat predicted differently according to gender: a lower risk of acute myocardial infarction in women and a higher risk in men [34]. Another study with obese youth confirmed harmful effects of android fat distribution on insulin resistance [35]. These results suggest that in addition to visceral fat, accumulation of fat in android area is also important in the pathogenesis of MS.

Of note, in this study, android fat was more closely associated with a clustering of metabolic abnormalities than visceral fat. There is no clear answer for this but several explanations can be postulated. First, android area defined in this study includes liver, pancreas and lower part of the heart. Many studies have shown that fat accumulation in these structures have more detrimental metabolic impacts through direct and indirect mechanisms [36]−[39]. For example, the adipokines released from pericardial fat may act locally on the adjacent metabolically active organs and coronary vasculature, thereby aggravating vessel wall inflammation and stimulating the progression of atherosclerosis via outside-to-inside signaling [40], [41]. Furthermore, fat accumulation in liver correlates more strongly with insulin sensitivity than visceral fat via adipocytokine signaling and/or low-grade inflammation mechanism [37], [39].

Second, the android fat represents whole fat amount in the upper abdomen area while VAT measurement was performed at a single umbilicus level. This different methodology may possibly contribute to greater association between metabolic impairments and android fat than VAT. This interpretation is supported by the borderline significance of VAT in the association with MS when combined VAT area was used instead of a single level of VAT. A recent study also showed that the whole fat amount between L1–L5 vertebra showed a stronger relationship with insulin resistance than that of the single L3 level [39].

In this study, both android fat amount and VAT were associated with coronary artery stenosis. Android fat is closely related with VAT because of their proximity and correlation with various cardiovascular risk factors. The attenuated associations of both variables without statistical significance in the regression model where android fat and VAT were simultaneously included may be due to a shared systemic effect as a result of shared risk factors for the development of atherosclerosis.

This study has several strengths. First, DXA with its advanced technology was used to measure regional fat depot. Second, the subjects were recruited from a well-defined population, which represented a single ethnic group and were older than 65 years. Third, the regression analysis was adjusted for important factors including whole body fat mass, insulin resistance, and biochemical markers including adiponectin and hsCRP that might affect MS.

This study also has several limitations. First, since our study is limited by its cross-sectional nature, it is impossible to confirm clinically meaningful role of android fat depot. Therefore, further studies are needed to determine a predictive role of android fat for a clustering of cardiometabolic risk factors and subsequent incidence of cardiovascular diseases. Second, this is a single cohort study with a small number of subjects and the results are confined to this specific cohort.

### Conclusion

Of the various body compositions examined using advanced techniques, android fat measured by DXA was significantly associated with clustering of five components of MS even after accounting for various factors including visceral adiposity. It would be interesting to apply this concept of body composition phenotypes to health risks in light of race/ethnic and age variability in metabolic susceptibility to obesity and MS. Further studies are necessary to determine whether the information gathered in the present study is generalizable to other populations and also to validate the practicality and implication of using android fat/DXA in predicting for cardiovascular diseases.

## Supporting Information

Table S1 **Participants characteristics including body composition measured by dual energy x-ray absorptiometry (DXA) and computed tomography (CT) subdivided by sex.**
(DOC)

Table S2 **Correlation between summation of components of metabolic syndrome and multiple parameters including body composition.**
(DOC)

Table S3 **Multivariate linear regression analysis of associations of multiple parameters including body composition with summation of five individual components of metabolic syndrome (VAT from L3-4 to L5-S1 was used).**
(DOC)
