# Supplementary material for: Android Fat Depot Is More Closely Associated with Metabolic Syndrome than Abdominal Visceral Fat in Elderly People
Source: PLoS One. 2011 Nov 11;6(11):e27694. doi: 10.1371/journal.pone.0027694 (PMC3214067; doi:10.1371/journal.pone.0027694)
Supplement: Table S1 — Participants characteristics including body composition measured by dual energy x-ray absorptiometry (DXA) and computed tomography (CT) subdivided by sex. (DOC) [file pone.0027694.s001.doc]

| **Table S1. Participants characteristics including body composition measured by dual energy x-ray absorptiometry (DXA) and computed tomography (CT) subdivided by sex** | | | | | |
| --- | --- | --- | --- | --- | --- |
|  | Male (n = 287) | | Female (n = 278) | | P-value |
|  | Mean | SD | Mean | SD |
| Age (years) | 73.6 | 7.6 | 72.5 | 6.7 | 0.077 |
| SBP (mmHg) | 83.3 | 10.6 | 83.5 | 11.0 | 0.997 |
| DBP (mmHg) | 164.9 | 6.0 | 150.8 | 5.6 | 0.818 |
| Height (cm) | 164.9 | 6.0 | 150.8 | 5.6 | < 0.001 |
| Weight (kg) | 65.5 | 9.8 | 56.2 | 8.4 | 0.034 |
| BMI (kg/m^2^) | 24.1 | 3.2 | 24.6 | 3.1 | 0.007 |
| Waist circumference (cm) | 88.0 | 8.6 | 85.7 | 9.6 | 0.007 |
| Smoking |  |  |  |  | 0.037 |
| Current smoker (n, %) | 59 | 20.6% | 10 | 3.6% | < 0.001 |
| Ex-smoker (n, %) | 158 | 55.1% | 7 | 2.5% |  |
| Never smoker (n, %) | 70 | 24.4% | 261 | 93.9% |  |
| Alcohol |  |  |  |  | < 0.001 |
| Current drinker (n, %) | 135 | 47.2% | 20 | 7.2% |  |
| Ex-drinker (n, %) | 65 | 22.7% | 20 | 7.2% |  |
| Never drinker (n, %) | 86 | 30.1% | 322 | 57.3% |  |
| Regular exercise (n, %) | 207 | 72.1% | 119 | 43.8% | < 0.001 |
| **By DXA** |  |  |  |  |  |
| Whole body muscle mass (kg) | 43.0 | 5.1 | 30.7 | 3.5 | < 0.001 |
| Whole body fat mass (kg) | 19.2 | 7.5 | 24.0 | 6.9 | < 0.001 |
| Android fat (kg) | 1.8 | 0.5 | 2.0 | 0.6 | 0.006 |
| Gynoid fat (kg) | 2.5 | 0.8 | 3.3 | 0.8 | < 0.001 |
| **By CT** |  |  |  |  |  |
| Visceral adipose tissue (cm^2^) | 131.4 | 65.5 | 120.0 | 46.7 | 0.046 |
| Subcutaneous adipose tissue (cm^2^) | 126.9 | 55.2 | 211.8 | 65.9 | < 0.001 |
| **Biochemical data** |  |  |  |  |  |
| Triglycerides (mg/dL) | 135.7 | 95.7 | 143.5 | 83.0 | 0.301 |
| HDL-cholesterol (mg/dL) | 43.9 | 12.1 | 47.2 | 12.5 | 0.002 |
| LDL-cholesterol (mg/dL) | 122.7 | 35.0 | 134.0 | 33.6 | < 0.001 |
| Fasting glucose (mg/dL) | 114.9 | 27.4 | 108.1 | 23.8 | 0.002 |
| Fasting insulin (μIU/mL) | 5.0 | 2.9 | 5.5 | 3.5 | 0.064 |
| HOMA-IR | 1.4 | 1.0 | 1.5 | 1.0 | 0.490 |
| A1C (%) | 6.2 | 0.9 | 6.1 | 0.8 | 0.250 |
| Adiponectin (μg/mL) | 7.9 | 5.3 | 9.4 | 6.1 | 0.001 |
| hsCRP (mg/dL) | 0.3 | 0.9 | 0.2 | 0.4 | 0.108 |
