# Supplementary material for: Android Fat Depot Is More Closely Associated with Metabolic Syndrome than Abdominal Visceral Fat in Elderly People
Source: PLoS One. 2011 Nov 11;6(11):e27694. doi: 10.1371/journal.pone.0027694 (PMC3214067; doi:10.1371/journal.pone.0027694)
Supplement: Table S2 — Correlation between summation of components of metabolic syndrome and multiple parameters including body composition. (DOC) [file pone.0027694.s002.doc]

| **Table S2. Correlation between summation of components of metabolic syndrome and multiple parameters including body composition** | | |
| --- | --- | --- |
|  | Summation of five components of metabolic syndrome | |
|  | *r* | P-value |
| Age (years) | 0.054 | 0.196 |
| BMI (kg/m^2^) | 0.425 | < 0.001 |
| Whole body muscle mass (kg) | 0.001 | 0.994 |
| Whole body fat mass (kg) | 0.423 | < 0.001 |
| Android fat (kg) | 0.460 | < 0.001 |
| Gynoid fat (kg) | 0.328 | < 0.001 |
| Visceral adipose tissue area (cm^2^) | 0.419 | < 0.001 |
| Subcutaneous adipose tissue area (cm^2^) | 0.409 | < 0.001 |
| Creatinine (mg/dL) | -0.024 | 0.575 |
| Alanine aminotransferase (IU/L) | 0.145 | 0.001 |
| γ-glutamyl transpeptidase (IU/L) | 0.102 | 0.015 |
| Total cholesterol (mg/dL) | 0.029 | 0.496 |
| LDL-cholesterol (mg/dL) | -0.041 | 0.330 |
| Insulin (μIU/mL) | 0.334 | < 0.001 |
| HOMA-IR | 0.403 | < 0.001 |
| Adiponectin (μg/mL) | -0.298 | < 0.001 |
| hsCRP (mg/dL) | -0.039 | 0.356 |
| HOMA-IR: homeostasis model assessment for insulin resistance | | |
