# Supplementary material for: Android Fat Depot Is More Closely Associated with Metabolic Syndrome than Abdominal Visceral Fat in Elderly People
Source: PLoS One. 2011 Nov 11;6(11):e27694. doi: 10.1371/journal.pone.0027694 (PMC3214067; doi:10.1371/journal.pone.0027694)
Supplement: Table S3 — Multivariate linear regression analysis of associations of multiple parameters including body composition with summation of five individual components of metabolic syndrome (VAT from L3-4 to L5-S1 was used). (DOC) [file pone.0027694.s003.doc]

| **Table S3. Multivariate linear regression analysis of associations of multiple parameters including body composition with summation of five individual components of metabolic syndrome (VAT from L3-4 to L5-S1 was used)** | | | |
| --- | --- | --- | --- |
|  | β coefficient | t | P-value |
| Model 1: Age, gender, smoking, exercise, BMI, hsCRP, LDL-cholesterol, adiponectin, HOMA-IR, and whole body fat mass adjusted | | | |
| Age (years) | 0.150 | 4.136 | < 0.001 |
| Gender (male vs. female) | 0.210 | 4.229 | < 0.001 |
| BMI (kg/m^2^) | 0.211 | 3.429 | 0.001 |
| hsCRP (≥ 2.5 mg/l vs. < 2.5 mg/l) | 0.187 | 3.012 | 0.034 |
| Adiponectin (μg/mL) | -0.225 | -6.002 | < 0.001 |
| HOMA-IR | 0.200 | 4.850 | < 0.001 |
| Whole body fat mass (kg) | 0.114 | 1809 | 0.071 |
| Model 2: Model 1 + VAT from L3-4 to L5-S1 | | | |
| Age (years) | 0.118 | 2.546 | 0.005 |
| Gender (male vs. female) | 0.265 | 4.212 | < 0.001 |
| BMI (kg/m^2^) | 0.156 | 1.893 | 0.060 |
| hsCRP (≥ 2.5 mg/l vs. < 2.5 mg/l) | 0.153 | 2.875 | 0.040 |
| Adiponectin (μg/mL) | -0.218 | -4.756 | < 0.001 |
| HOMA-IR | 0.182 | 3.2092 | 0.008 |
| VAT (cm^2^) from L3-4 to L5-S1 | 0.172 | 2.493 | 0.013 |
| Model 3: Model 1 + android fat | | | |
| Age (years) | 0.143 | 3.965 | < 0.001 |
| Gender (male vs. female) | 0.275 | 5.204 | < 0.001 |
| BMI (kg/m^2^) | 0.207 | 3.399 | 0.001 |
| hsCRP (≥ 2.5 mg/l vs. < 2.5 mg/l) | 0.142 | 2.528 | 0.063 |
| Adiponectin (μg/mL) | -0.194 | -5.094 | < 0.001 |
| HOMA-IR | 0.173 | 4.153 | < 0.001 |
| Whole body fat mass (kg) | -0.243 | -1.976 | 0.049 |
| Android fat (kg) | 0.384 | 3.381 | 0.001 |
| Model 4: Model 1 + VAT from L3-4 to L5-S1 + android fat | | | |
| Age (years) | 0.119 | 2.712 | 0.007 |
| Gender (male vs. female) | 0.317 | 5.032 | < 0.001 |
| BMI (kg/m^2^) | 0.151 | 1.976 | 0.049 |
| Adiponectin (μg/mL) | -0.203 | -4.298 | < 0.001 |
| HOMA-IR | 0.159 | 3.043 | 0.003 |
| VAT (cm^2^) from L3-4 to L5-S1 | 0.145 | 1.723 | 0.082 |
| Android fat (kg) | 0.378 | 2.404 | 0.017 |
| HOMA-IR: homeostasis model assessment for insulin resistance | | | |
